# Analysis of the Risk Factors for Elevated D-Dimer Level After Breast Cancer Surgery: A Multicenter Study Based on Nursing Follow-Up Data

**DOI:** 10.3389/fonc.2022.772726

**Published:** 2022-07-19

**Authors:** Yanqiu Wang, Xi Liang, Shujun Wang, Yuying Wang, Ling Qin, Danni Chen, Yanlin Jiang, Hao Zhang

**Affiliations:** ^1^ Department of Breast Surgery, Second Hospital of Dalian Medical University, Dalian, China; ^2^ Department of Obstetrics and Gynecology, Second Hospital of Dalian Medical University, Dalian, China; ^3^ Department of Breast Surgery, Cancer Hospital of China Medical University, Liaoning Cancer Hospital and Institute, Shenyang, China; ^4^ Department of Operation Room, Second Hospital of Dalian Medical University, Dalian, China; ^5^ Department of Neurology, Boao Yiling Life Care Center, Boao, China; ^6^ Department of Breast Surgery, Affiliated Zhongshan Hospital of Dalian University, Dalian, China

**Keywords:** D-dimer, breast cancer, surgery, nursing, thrombosis

## Abstract

D-dimer level is often used to assess the severity of trauma as well as the risk of thrombosis. This study investigated the risk factors for high postoperative D-dimer level. This study included a total of 2706 patients undergoing breast cancer surgery to examine the associations between various clinicopathological factors and variation in D-dimer levels. After adjusting for other factors, T stage, neoadjuvant chemotherapy, blood loss, surgery type, diabetes, and elevated leukocyte and neutrophil counts were found to be significant risk factors for D-dimer variation. This study identified several factors associated with elevated D-dimer levels and consequent thrombosis after breast cancer surgery, which may aid in the development of more precise preventive measures and interventions as well as serve as a reference for future research.

## Introduction

Breast cancer has recently become the most common malignancy worldwide and is also the leading type of cancer in China. With advances in treatment techniques, relatively good therapeutic effects are generally achieved in the treatment of breast cancer (1, 2). Therefore, good survival rates and quality of life are expected in early-stage patients (3, 4). There are relatively few severe complications in the perioperative period of breast cancer surgery, with the most severe being venous thrombosis of the lower extremities and the consequent pulmonary embolism, which often lead to serious damage to health or death of patients and a heavy financial burden (5). In general, a smaller scope of surgery can effectively reduce the occurrence of complications and is indicative of better postoperative breast appearance (6–8). Therefore, breast-conserving surgery has become an increasingly popular approach among patients who meet the relevant indications. The reduced degree of injury provides several benefits, including fewer surgical complications, faster recovery, and increased long-term survival (9–11). However, these methods pose new challenges for the postoperative care of patients with breast cancer. Thus, a comprehensive analysis of the clinicopathological features and surgical approach of patients is required to assess the degree of trauma and risk of thrombosis.

To maintain normal physiological status, the coagulation system is activated to prevent blood loss in the event of vascular injury. Despite its lack of specificity, an elevated D-dimer level serves as a relatively sensitive biomarker for thrombosis in the circulatory system. Therefore, D-dimer level is often utilized in clinical practice to aid in the diagnosis of venous thromboembolism, deep vein thrombosis, and pulmonary embolism. D-dimer level has also been shown to correlate directly with the degree of trauma, making it an appropriate indicator for assessing injury severity (12, 13). Therefore, when we use D-dimer level as an index to measure the risk of thrombosis, we should realize the limitations of D-dimer as a negative predictive factor for adverse events and pay attention to excluding other influencing factors, such as trauma scope, age, preoperative treatment, disease stage, etc., and analyze the independent risk of different factors under the coexistence of multiple factors. At the same time, in the era of epidemic, the treatment of tumor also has some new challenges. Have patients with COVID-19 affected the coagulation of blood because of pathological changes in the lungs? (14) Whether or not you have ever suffered from COVID-19, will the vaccination affect the hemagglutination state? (15) For tumor patients, their own diseases may bring blood hypercoagulability. At the same time, tumor patients also have the characteristics of relatively old age. In these patients, how to measure the relationship between coagulation indexes and thrombosis risk is also a subject that needs special analysis (16, 17). In order to more accurately understand the potential risk of thrombosis in patients, we should also continuously refine the monitoring process of D-dimer, find the most accurate time point for blood sampling and testing, and develop more biomarkers for modeling and prediction, so as to avoid the influence of multiple confounding factors caused by simple D-dimer (18–21). In a word, under the situation of epidemic situation, aging population and increasing tumor incidence rate, how to give consideration to the treatment effect of tumor and the quality of life of patients, and maximize the net benefit of patients is a subject that needs to be continuously studied. In this context, the present study investigated the role of D-dimer level in thrombosis and injury before and after surgery for the treatment of breast cancer. We aimed to explore independent risk factors for elevated postoperative D-dimer level and the influencing factors of the degree of trauma and risk of thrombosis to provide a basis for the identification of patients who require close monitoring.

## Methods

### Patients

This study enrolled 2706 patients who underwent breast cancer surgery from 2013-2020, including total mastectomy (glandectomy), and breast-conserving surgery, at the Second Hospital of Dalian Medical University, and the Affiliated Zhongshan Hospital of Dalian University. The inclusion criteria were: 1. The patient was diagnosed with breast cancer and underwent breast cancer surgery, and the time from the last general anesthesia operation was more than one year; 2. Received or not neoadjuvant therapy before operation; 3. 18-75 years old; 4. No distant metastasis; 5. Complete clinicopathological information, especially the test results of D-dimer before and after operation. The exclusion criteria were: 1. Received general anesthesia within one year; 2. The patient was younger than 18 years old or older than 75 years old; 3. There were distant metastatic lesions before operation; 4. The clinicopathological information is incomplete. The clinical, surgical, and pathological findings and all medical data, including age, tumor stage, nodal stage, neoadjuvant chemotherapy, operative time, blood loss, surgery, preoperative and postoperative complications were collected prospectively and recorded in a database.

This study was approved by the Ethics Committee of Second Hospital of Dalian Medical University. All methods were performed in accordance with the relevant guidelines and regulations. Informed consent was obtained from all participants.

### Blood Sampling

Blood samples for the determination of pre- and postoperative D-dimer levels were acquired together with the samples used for other hematological tests. Preoperative sampling was usually performed on the second day after admission, approximately 3 days before surgery. Before surgery, in addition to D-dimer, we will detect the patient’s blood routine, liver function, renal function, coagulation, blood glucose, blood lipid, blood ions, etc. Postoperative sampling was usually performed 1 day after surgery. After the operation, we will detect the blood routine, blood ions, four items of coagulation, D-dimer, etc.

### Statistical Analyses

Chi-square tests were used to analyze the differences between groups of various D-dimer levels. Correlation analyses were used to identify the influencing factors of D-dimer levels. Multivariate analyses using the ENTER method were conducted to assess the risk factors for increased D-dimer levels. All analyses were performed using IBM SPSS Statistics for Windows, version 23.0. Two-sided P-values <0.05 were considered statistically significant.

## Results

### Patient Characteristics

A total of 13 clinicopathological factors were identified and included in the analysis. The distribution of various clinicopathological factors was subsequently compared between the two groups (pre- and postoperative D-dimer levels). Of these, T stage, neoadjuvant chemotherapy, blood loss, surgery, diabetes, and leukocyte and neutrophil elevation differed significantly between the two D-dimer groups (**Table 1**). The factors with significantly different distributions between the two groups were identified as possible risk factors for D-dimer variation.

**Table 1:** Characteristics of population by D-dimer increase level (n = 2706).

Characteristics	≤150	>150	p Value
**Age (years)**			0.054
≤60	364 (39)	749 (43)	
>60	578 (61)	1015 (57)	
**Pathological tumour stage** (%)			<0.001
T1	73 (8)	95 (5)	
T2	136 (14)	171 (10)	
T3	433 (46)	920 (52)	
T4	300 (32)	578 (33)	
**Pathological nodal stage** (%)			0.249
N0	574 (61)	1100 (62)	
N1	249 (26)	486 (28)	
N2	85 (9)	126 (7)	
N3	34 (4)	52 (3)	
**Neoadjuvant chemotherapy (%)**			<0.001
No	635 (67)	966 (55)	
Yes	307 (33)	798 (45)	
**Operative time** (%)			0.103
<2h	216 (23)	357 (20)	
>2h	726 (77)	1407 (80)	
**Blood loss**			<0.001
<100 ml	588 (72)	914 (62)	
>100 ml	233 (28)	567 (38)	
**Surgery**			<0.001
Total mastectomy	643 (68)	929 (53)	
BCS + SLNB	299 (31)	835 (47)	
**Preoperative co-morbidities**			
Diabetes	84 (9)	308 (18)	<0.001
Hypertension	272 (29)	490 (28)	0.546
Cardiac history	62 (7)	123 (7)	0.701
Cerebrovascular disease	53 (6)	141 (8)	0.023
**Postoperative complications**			
Leukocyte elevation	49 (5)	145 (8)	0.004
Neutrophil elevation	42 (5)	170 (10)	<0.001

### Correlation Analysis

Spearman correlation analysis showed linear correlations between D-dimer difference and neoadjuvant chemotherapy, T stage, surgery, blood loss, diabetes, leukocyte and neutrophil elevation, and cerebrovascular disease (**Table 2**).

**Table 2:** Spearman correlation analysis between clinicopathological features and D-dimer.

Clinicopathological features	D-dimer ( p; Spearman correlation)
Neoadjuvant chemotherapy	<0.001 (0.123)
T stage	0.01 (0.05)
Surgery	<0.001 (0.151)
Blood loss	<0.001 (0.100)
Diabetes	<0.001 (0.116)
Leukocyte elevation	0.004 (0.056)
Neutrophil elevation	<0.001 (0.092)
Cerebrovascular disease	0.023 (0.044)

### Risk Factor Analysis

The associations between the possible risk factors and elevated D-dimer level is shown in **Table 3**. After adjusting for the 13 variables, T stage, neoadjuvant chemotherapy, blood loss, surgery type, diabetes, and leukocyte and neutrophil elevation were identified as significant risk factors.

**Table 3:** OR for increasing of D-dimer—multivariable analysis (n = 623).

	Enter method	
	OR (95% CI)	p
**Age**	0.906 (0.728-1.127)	0.374
**Pathological tumor stage**		0.002
T1	Ref	
T2	1.024 (0.624-1.679)	0.926
T3	1.525 (0.962-2.417)	0.073
T4	1.813 (1.095-3.004)	0.021
**Pathological nodal stage**		0.926
N0	Ref	
N1	0.967 (0.762-1.226)	0.781
N2	0.868 (0.613-1.230)	0.425
N3	1.141 (0.626-2.077)	0.667
**Neoadjuvant chemotherapy**	2.251 (1.811-2.797)	<0.001
**Operative time**	0.849 (0.675-1.068)	0.162
**Blood loss**	1.686 (1.375-2.067)	<0.001
**Surgery**	1.676 (1.350-2.082)	<0.001
**Preoperative complications**		
Diabetes	2.587 (1.933-3.461)	<0.001
Hypertension	0.928 (0.732-1.175)	0.533
Cardiac history	0.852 (0.601-1.208)	0.369
Cerebrovascular disease	1.413 (0.961-2.078)	0.079
**Postoperative complications**		
Leukocyte elevation	1.840 (1.247-2.715)	0.002
Neutrophil elevation	3.364 (2.159-5.241)	<0.001

## Discussion

In recent years, increasing attention has been paid to the concept of fast-track surgery, which aims to provide postoperative patients with a variety of integrated treatment approaches to achieve rapid recovery. This leads to the reduction of psychological and organic traumatic stress reactions, which ultimately reduces postoperative complications, shortens the average length of hospital stay, decreases the risk of death, and reduces health care costs (22). However, fast-track surgery is not yet optimized and remains under development. Modifications are also required for specific treatment measures to enhance their suitability for the Chinese population. Therefore, clinicopathological data, surgical information, postoperative complication data, and the prognostic and follow-up information of patients requiring surgery must be collected continuously to perform statistical analysis to aid the development of more reliable and effective diagnostic and treatment modalities for the Chinese population (23, 24).

The present study identified T stage, neoadjuvant chemotherapy, blood loss, surgery type, diabetes, and leukocyte and neutrophil elevation as risk factors for significantly increased D-dimer level.

Our results differ from those of previous studies in that age was not an independent risk factor. Although this is contradictory to the conventional belief that older age is associated with a higher risk of concomitant thrombosis, it suggests that younger patients should also be monitored closely for the occurrence of postoperative venous thrombosis (25–29).

With the increase in the proportion of patients undergoing neoadjuvant chemotherapy, there is a greater need to closely monitor D-dimer level and the possible risk of venous thrombosis (20). During surgery, efforts should also be made to minimize operative time and reduce intraoperative bleeding. For patients in whom the breast and axilla can be preserved, the scope of surgery should also be minimized. To a certain extent, D-dimer level can reflect the degree of damage to the body. Previous studies did not report surgical approach as having a significant effect on D-dimer level; however, its effects have gradually become pronounced with the increasing number of cases (30–32). This may be attributed to the presence of confounding bias among surgical approaches in previous studies, such as the prolonged operative time and intraoperative pathological waiting time for breast-conserving surgery and sentinel lymph node biopsy. Operative time was also not an independent factor due to various confounding factors.

Diabetes mellitus is a known risk factor for thrombosis (33, 34). In particular, the physiological changes following general anesthesia often result in high blood glucose levels, which increase the risk of thrombosis when combined with prolonged bed rest. Thus, careful patient monitoring is required.

Postoperative infection, especially cellular infection that results in elevated neutrophil count, is a risk factor for thrombosis (35–37). It may also exacerbate injury and lead to elevated D-dimer levels. Although the degree of D-dimer elevation does not necessarily correlate with the risk of thrombosis and degree of physical injury, measures should still be adopted to prevent postoperative infections such as drain-related, wound, and urinary tract infections.

A high postoperative D-dimer level may correspond to more severe physical injury and a higher risk of postoperative thrombosis. Our findings indicated that, despite advances in diagnosis and treatment, it is still critical for researchers to collect data on basic patient information, records of previous treatment, and the prevention and treatment of perioperative complications.

## Conclusions

The results of the present study revealed multiple risk factors that may cause a significant increase in D-dimer level in the postoperative period. These findings suggest the need to pay particular attention to these patients during the perioperative period, adopt adequate preventive measures, and conduct relevant research.

## Data Availability Statement

The raw data supporting the conclusions of this article will be made available by the authors, without undue reservation.

## Ethics Statement

This study was reviewed and approved by Second Hospital of Dalian Medical University. The patients/participants provided their written informed consent to participate in this study. Written informed consent was obtained from the individual(s) for the publication of any potentially identifiable images or data included in this article.

## Author Contributions

LQ, YQW, YYW and XL participated in the study design and manuscript drafting. SW, HZ, and YJ participated in the study design and manuscript drafting. DC, HZ, and YJ participated in the statistical analysis and manuscript drafting. All authors contributed to the article and approved the submitted version.

## Funding

This research was funded by Dalian Medical Science Research Project (1612023).

## Conflict of Interest

The authors declare that the research was conducted in the absence of any commercial or financial relationships that could be construed as a potential conflict of interest.

## Publisher’s Note

All claims expressed in this article are solely those of the authors and do not necessarily represent those of their affiliated organizations, or those of the publisher, the editors and the reviewers. Any product that may be evaluated in this article, or claim that may be made by its manufacturer, is not guaranteed or endorsed by the publisher.
